# Serological Evidence of Influenza A Viruses in Frugivorous Bats from Africa

**DOI:** 10.1371/journal.pone.0127035

**Published:** 2015-05-12

**Authors:** Gudrun Stephanie Freidl, Tabea Binger, Marcel Alexander Müller, Erwin de Bruin, Janko van Beek, Victor Max Corman, Andrea Rasche, Jan Felix Drexler, Augustina Sylverken, Samuel K. Oppong, Yaw Adu-Sarkodie, Marco Tschapka, Veronika M. Cottontail, Christian Drosten, Marion Koopmans

**Affiliations:** 1 Center for Infectious Diseases Research, Diagnostics and Screening, Department of Virology, National Institute for Public Health and the Environment, Bilthoven, the Netherlands; 2 Viroscience Department, Erasmus Medical Center, Rotterdam, the Netherlands; 3 Institute of Virology, University of Bonn Medical Centre, Bonn, Germany; 4 Kumasi Center for Collaborative Research in Tropical Medicine, Kumasi, Ghana; 5 Kwame Nkrumah University of Science and Technology, Kumasi, Ghana; 6 Institute of Evolutionary Ecology and Conservation Genomics, University of Ulm, Ulm, Germany; 7 Smithsonian Tropical Research Institute, Balboa, Panama; CSIRO, AUSTRALIA

## Abstract

Bats are likely natural hosts for a range of zoonotic viruses such as Marburg, Ebola, Rabies, as well as for various Corona- and Paramyxoviruses. In 2009/10, researchers discovered RNA of two novel influenza virus subtypes – H17N10 and H18N11 – in Central and South American fruit bats. The identification of bats as possible additional reservoir for influenza A viruses raises questions about the role of this mammalian taxon in influenza A virus ecology and possible public health relevance. As molecular testing can be limited by a short time window in which the virus is present, serological testing provides information about past infections and virus spread in populations after the virus has been cleared. This study aimed at screening available sera from 100 free-ranging, frugivorous bats (*Eidolon helvum*) sampled in 2009/10 in Ghana, for the presence of antibodies against the complete panel of influenza A haemagglutinin (HA) types ranging from H1 to H18 by means of a protein microarray platform. This technique enables simultaneous serological testing against multiple recombinant HA-types in 5μl of serum. Preliminary results indicate serological evidence against avian influenza subtype H9 in about 30% of the animals screened, with low-level cross-reactivity to phylogenetically closely related subtypes H8 and H12. To our knowledge, this is the first report of serological evidence of influenza A viruses other than H17 and H18 in bats. As avian influenza subtype H9 is associated with human infections, the implications of our findings from a public health context remain to be investigated.

## Introduction

Bats are likely reservoirs for a range of zoonotic viruses, such as rabies and other lyssaviruses (family *Rhabdoviridae*), Ebola- and Marburg viruses (*Filoviridae*), Hendra- and Nipah viruses (*Paramyxoviridae*), as well as severe acute respiratory syndrome (SARS) virus (*Coronaviridae*) [1]. In 2009/10, influenza A expanded the list of viral pathogens found in bats, when RNA of two novel influenza A virus (IAV) subtypes (*Orthomyxoviridae*), H17N10 and H18N11, was discovered in frugivorous bats from Guatemala and Peru, respectively [2,3]. Until then, sixteen hemagglutinin (HA)- and nine neuraminidase (NA) types, two surface proteins utilized to classify IAV into subtypes, had been previously described. Water- and shore birds have been known to be the only relevant reservoir hosts for IAV [4]. Several IAV subtypes originating from birds have established stable lineages in birds, pigs and humans. Other avian (e.g., H5N1, H6N2, H7N9, H10N8) and swine influenza virus subtypes (e.g., most recently H3N2v) occasionally cause human infection, resulting in mild- to severe disease and occasional death [5].

Although the two newly discovered subtypes were recently found to have no zoonotic potential [6], the aim of this study was to investigate the role of bats as potential mammalian reservoirs for possibly zoonotic influenza A viruses, by screening for serological evidence against all currently known influenza virus HA-types in frugivorous bats from Ghana.

## Methods

### Ethics statement

As described previously [7], all animals used in this study were captured and sampled with permission from the Wildlife Division, Forestry Commission, Accra, Ghana. Geographic coordinates of the sampling site in Kumasi/Ghana were N06°42´02.0´´ W001°37´29.9´´. Capturing was conducted under the auspices of Ghana authorities. Following anesthesia using a Ketamine/Xylazine mixture, skilled staff exsanguinated all bats (permit no. CHRPE49/09; A04957). Samples were exported under a state contract between the Republic of Ghana and the Federal Republic of Germany. An additional export permission was obtained from the Veterinary Services of the Ghana Ministry of Food and Agriculture (permit no. CHRPE49/09; A04957). Materials of all sacrificed animals were used for various studies [8–11].

### Sample Analysis

Serum samples (n = 100) from straw-colored fruit bats (*Eidolon helvum*, Pteropodidae) were collected in 2009 (n = 81) and 2010 (n = 19) in Kumasi Zoo in Ghana. Although sampling was performed at Kumasi Zoo, all bats included in this study belonged to a wild, migratory colony roosting in trees on site at the time of sample collection.

For serological testing, we used a modification of the protein microarray (PA) technique as previously described by Koopmans et al. [12] and Freidl et al. [13]. 31 recombinant proteins of influenza A viruses [globular head domains (HA1)] were printed in duplicates onto nitrocellulose Film-slides (16 pad, ONCYTE AVID, Grace Bio-Labs, Bend, Oregon, USA). Selected proteins comprised various strains of all presently known influenza A virus HA-types. Reactivity and optimal working concentration of the proteins were determined by means of checkerboard titrations using specific rabbit antisera homologous to the antigens used (Table 1). Printed slides were stored in a desiccation chamber until further use.

**Table 1:** Recombinant HA1-proteins included in the protein microarray.

#	Code	Subtype	Strain
1	H1.18	H1N1	A/South Carolina/1/18
2	H1.77	H1N1	A/USSR/92/1977
3	H1.07	H1N1	A/Brisbane/59/2007
4	H1.09	H1N1	A/California/6/2009
5	H2.05	H2N2	A/Canada/720/05
6	H3.68	H3N2	A/Aichi/2/1968(H3N2)
7	H3.10	H3N2v	A/Minnesota/09/2010
8	H3.07	H3N2	A/Brisbane/10/2007
9	H4.02	H4N6	A/mallard/Ohio/657/2002
10	H5.97	H5N1	A/Hong Kong/156/97 (HP, clade 0) ^a^
11	H5.02	H5N8	A/duck/NY/191255-59/2002, LP ^b^
12	H5.10	H5N1	A/Hubei/1/2010 (HP, clade 2.3.2.1) ^a^
13	H5.06	H5N1	A/Turkey/15/2006 (HP, clade 2.2) ^a^
14	H5.07	H5N1	A/Cambodia/R0405050/2007 (HP, clade 1) ^a^
15	H5.05	H5N1	A/Anhui/1/2005 (HP, clade 2.3.4) ^a^
16	H6.07	H6N1	A/northern shoveler/California/HKWF115/2007
17	H7.03	H7N7	A/Chicken/Netherlands/1/03 (HP) ^a^
18	H7.13	H7N9	A/chicken/Anhui/1/2013 (LP)^b^
19	H7.12	H7N3	A/chicken/Jalisco/CPA1/2012 (HP)
20	H8.79	H8N4	A/pintail duck/Alberta/114/1979
21	H9.97	H9N2	A/chicken/Hong Kong/G9/97 (G9 lineage)
22	H9.09	H9N2	A/Hong-Kong/33982/2009 (G1 lineage)
23	H10.07	H10N7	A/blue-winged teal/Louisiana/Sg00073/07
24	H11.02	H11N2	A/duck/Yangzhou/906/2002
25	H12.91	H12N5	A/green-winged teal/ALB/199/1991
26	H13.00	H13N8	A/black-headed gull/Netherlands/1/00
27	H14.82	H14N5	A/mallard/Astrakhan/263/1982new
28	H15.83	H15N8	A/duck/AUS/341/1983
29	H16.99	H16N3	A/black-headed gull/Sweden/5/99
30	H17.09	H17N10	A/little yellow shouldered bat/ Guatemala/153/2009
31	H18.14	H18N11	A/flat-faced bat/Peru/033/2010

Proteins were obtained from Immune Technology Corp. (NY, USA) or Sino Biological Inc. (Beijing, China).

^a^highly pathogenic

^b^low pathogenic

Prior to analysis, we inactivated all bat sera in a water bath at 56°C for one hour. Four-fold sample dilutions were prepared in Blotto Blocking Buffer (Thermo Fisher Scientific Inc., Rockford, MA, USA) containing 0.1% Surfactant-Amps (Thermo Fisher Scientific Inc.). We used five microliters of serum for a starting concentration of 1:40.

Serum dilutions were incubated for one hour in a moist, dark box at 37°C. Antibody binding was detected using an unconjugated goat-anti-bat whole immunoglobulin G (IgG) (Bethyl Laboratories Inc., Montgomery, TX, USA) at a dilution of 1:800, in combination with a Fc-fragment specific AlexaFluor647-labelled rabbit-anti-goat IgG (Jackson ImmunoResearch Laboratories Inc, West Grove, PA, USA) at 1:1100. Both conjugates were titrated to determine the optimal working concentration prior to sample analysis. Each incubation period followed three washing steps using wash buffer (Maine Manufacturing, Maine, USA).

Fluorescent signals were measured with a Powerscanner (Tecan Group Ltd, Männedorf, Switzerland) and converted into titers as described before [12,13]. The detection capacity of the PA spanned a titer range from 40 to 2560. Samples showing no antibody reactivity were regarded as negative and were assigned a titer of 20 (half of the starting dilution). As we were unable to formally calculate a cut-off based on confirmed influenza A-positive and negative bat sera due to unavailability of such materials, an arbitrary cut-off of 40 was chosen, similarly to previous work [12]. Hence, samples displaying a dilution curve resulting in a titer of ≥40 were interpreted as positive. Comparisons of seropositivity between sexes, age groups, sampling seasons and-years, respectively, were performed using Chi^2^- or Fisher’s exact test in RStudio (Version 0.98.507, Boston, MA, USA) with a significance level of 0.05.

## Results

**Fig 1:**
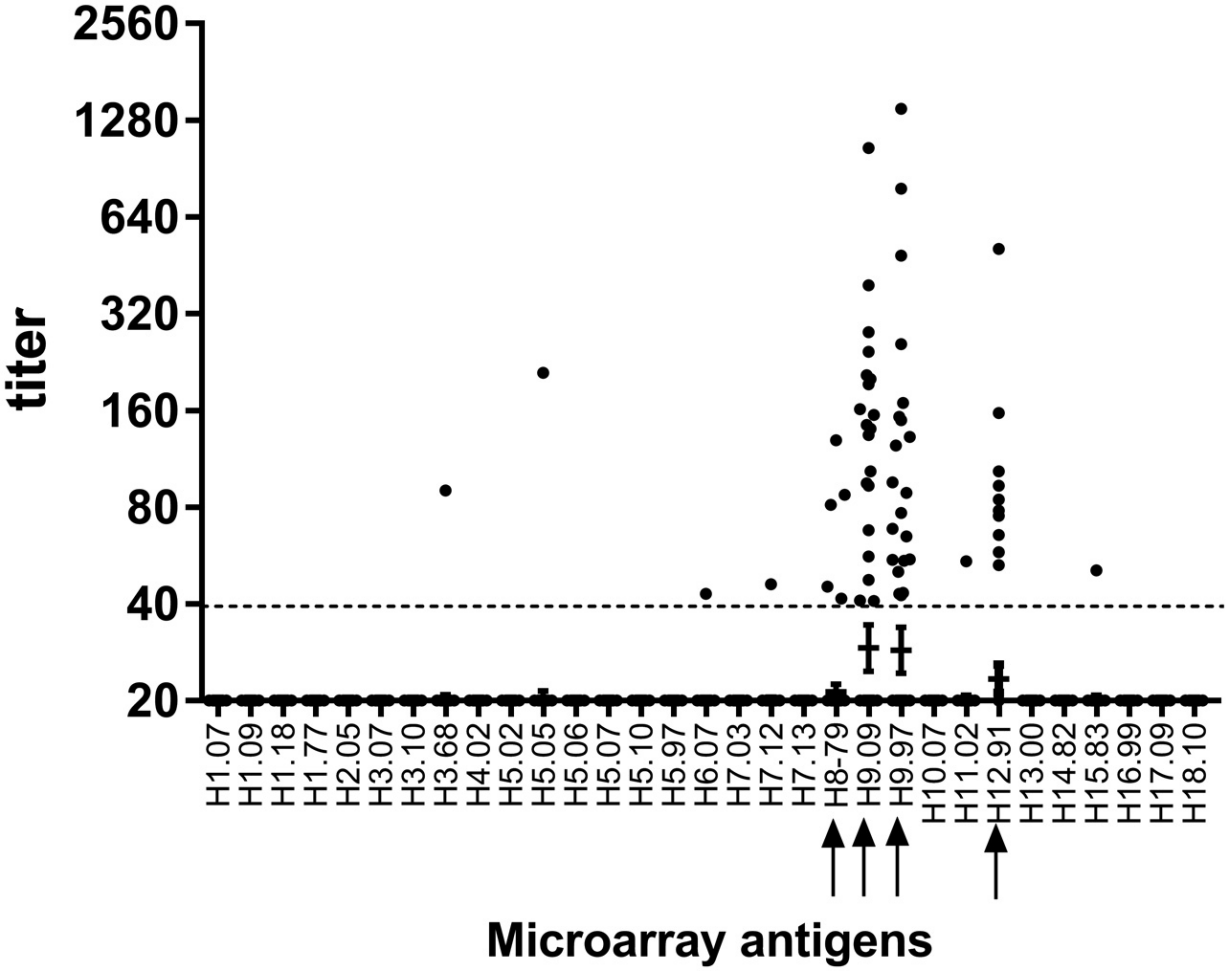
Titers of individual Ghanaian bats plotted against all recombinant proteins included on the microarray. Horizontal bars represent geometric mean titers per antigen including a 95% confidence interval. Sera below fluorescence values of 31.268 (half of the fluorescence spectrum) were regarded as negative and were assigned a titer of 20 (half of the starting dilution of 1:40). Sera above 40 (dashed line) were regarded as positive. Arrows indicate antigens grouping within the same phylogenetic cluster.

Of the 100 bats tested, 67 (67%) were negative against all antigens (Fig 1), whereas 33 bats showed titers against one or more antigens [2009: 26 (32%); 2010: 7 (37%)]. Four antigens showed slightly elevated geometric mean titers (GMT): H8.79: 21, H9.97: 29, H9.09: 29, H12.91: 23, whereas the GMT against the remaining antigens was 20 (Fig 1). Thirty bats (30%) showed antibody titers higher than 40 against at least one H9-antigen included on the PA. Thereof, 21 (21%) bats were positive for H9.97 (range: 43–1388) and 20 (20%) for H9.09 (range: 41–1048), respectively. Eleven animals showed antibody reactivity against both H9-antigens (11%), whereas ten individuals (10%) selectively reacted with H9.97, and nine (9%) solely with H9.09. Of the H9-positive bats, 24 were sampled in 2009 (29.6%) and 6 in 2010 (31.6%), respectively.

Ten of the samples from H9 positive bats also bound to the H12.91 protein (range: 53–509), and four in addition bound to H8.79 protein (range: 42–129). Serum from one of these four bats had additional reactivity to H11.02 antigen. Similarly, two other H9.97-positive individuals, reacted against H7.12 (titer: 46) and H15.83 (51), or with H6.07 (43). Unique reactivity to single proteins was observed for H3 (n = 1), H5 (n = 1) and H8 (n = 1, titer: 42). We found no significant differences in seropositivity between sexes, age groups, sampling season and —year, respectively (Table 2).

**Table 2:** Serological findings versus sex, age group, sampling year and—season.

		serology			
		negative	positive	Row total	p-value
**sex** ^a^	male	51	22	73	0.4463
	female	16	11	27	
**age group** ^b^	adult	54	31	85	0.134
	juvenile	13	2	15	
**year** ^a^	2009	55	26	81	0.9008
	2010	12	7	19	
**season** ^b^ ^,^ ^c^	dry	6	4	10	0.7259
	rainy	61	29	90	
Column total		67	33	**100**	

Comparisons showed no significant differences. Counts also reflect percentages (n = 100).

^a^Pearson’s Chi^2^-test with continuity correction

^b^Fisher’s exact test

^c^dry: December to February, rainy: March to July and September to November.

## Discussion and Conclusions

In this study, we report on serological evidence of influenza A viruses in straw-colored fruit bats from Ghana. We found no reactivity against H17 antigens such as recorded in bats from Guatemala (38% [2] or H18 antigens like in bats from Peru (27% [3]. However, we studied a not closely related bat species from a different continent, and found about 30% antibody detection rate against HA-type H9. Sonntag et al. [14] and Fereidouni et al. [15] screened Central European bats for genomic traces of influenza virus using generic RT-PCR assays but found no such evidence. For the Central and South American bats influenza virus RNA was detected in a low percentage (0.9%) of the Guatemalan (3/316) and Peruvian (1/110) bats by pan-influenza conventional RT-PCR [2,3]. However, molecular detection is limited by a short duration of virus excretion making it impossible to exclude virus presence on population level. Serological techniques can shed light on the infection history even after the virus has been cleared by the immune system. Still, serological tests are limited in their specificity due to the existence of cross reactive antibodies [16]. As with every attempt to study infection in novel host systems, our techniques are not finally optimized for use with bats due to limited availability of reagents (including confirmed influenza A positive- and negative bat sera) [17,18]. Moreover, there was insufficient sample material for additional analyses such as microneutralization- or hemagglutination inhibition assays due to utilization of materials in prior studies [8–11]. Our results are preliminary in this regard, leaving the possibility that antibodies in bats may not be directed against typical avian-origin H9 HA lineages, but outlier viruses yet to be discovered.

Nevertheless, several of the bat sera showing H9-reactivity also reacted with antigens H8 and H12. This supports the credibility of our findings as there is clear phylogenetic relatedness of these particular subtypes[19]. Similar intra-clade reactivity was previously observed in chickens naturally infected with subtype H9N2 [13]. No significant association in seropositivity between sex, age groups, sampling year and season, respectively, could be found. However, as sample size was small we cannot rule out that potentially significant associations might have been missed.

Influenza A viruses of HA-type H9 have a wide geographical distribution in birds and are recognized as possible candidates to cause a future pandemic [20]. In addition, this subtype is associated with human infection causing mostly mild symptoms, which likely leads to an underestimation of cases [5]. Limited surveillance in birds allow unnoticed reassortment events between circulating avian or potentially human influenza virus strains, resulting in variants with yet unknown zoonotic potential [21]. Given the relatively high seroprevalence found in bats in both sampling years and the clinically healthy status at the time of sample collection, we cautiously suggest that bats—as for other emerging viruses [22]—might constitute asymptomatic mammalian carriers of influenza A viruses. In summary, we present serological evidence of influenza A viruses in Old World fruit bats that have been shown to be biologically relevant reservoirs of pathogenic viruses such as Henipaviruses, Coronaviruses, Lyssaviruses and Filoviruses. It is conceivable that there might be a link between serological evidence of influenza A virus in bats and migratory birds, as their flyways overlap with the geographic distribution of *E*. *helvum* [23,24]. However, in the absence of molecular data this hypothesis remains speculative. As *E*. *helvum* are widely consumed as bush meat in West Africa [25], the implications of the findings from a public health perspective remain to be investigated. Serological studies in humans consuming bats (including suitable control groups) would be useful to shed light on possible spillover events.
